# Hyaluronic Acid Present in the Tumor Microenvironment Can Negate the Pro-apototic Effect of a Recombinant Fragment of Human Surfactant Protein D on Breast Cancer Cells

**DOI:** 10.3389/fimmu.2020.01171

**Published:** 2020-07-08

**Authors:** Valarmathy Murugaiah, Chiara Agostinis, Praveen M. Varghese, Beatrice Belmonte, Salvatore Vieni, Fanan A. Alaql, Salman H. Alrokayan, Haseeb A. Khan, Anuvinder Kaur, Terry Roberts, Taruna Madan, Roberta Bulla, Uday Kishore

**Affiliations:** ^1^Biosciences, College of Health and Life Sciences, Brunel University London, Uxbridge, United Kingdom; ^2^Institute for Maternal and Child Health, Istituto di Ricovero e Cura a Carattere Scientifico (IRCCS) Burlo Garofolo, Trieste, Italy; ^3^School of Biosciences and Technology, Vellore Institute of Technology, Vellore, India; ^4^Tumor Immunology Unit, Human Pathology Section, Department of Health Sciences, University of Palermo, Palermo, Italy; ^5^Division of General and Oncological Surgery, Department of Surgical, Oncological and Oral Sciences, University of Palermo, Palermo, Italy; ^6^Department of Biochemistry, College of Science, King Saud University, Riyadh, Saudi Arabia; ^7^Department of Innate Immunity, ICMR—National Institute for Research in Reproductive Health, Mumbai, India; ^8^Department of Life Sciences, University of Trieste, Trieste, Italy

**Keywords:** innate immunity, surfactant protein D, immune surveillance, breast cancer, hyaluronic acid

## Abstract

Human surfactant protein D (SP-D) belongs to the family of collectins that is composed of a characteristic amino-terminal collagenous region and a carboxy-terminal C-type lectin domain. Being present at the mucosal surfaces, SP-D acts as a potent innate immune molecule and offers protection against non-self and altered self, such as pathogens, allergens, and tumor. Here, we examined the effect of a recombinant fragment of human SP-D (rfhSP-D) on a range of breast cancer lines. Breast cancer has four molecular subtypes characterized by varied expressions of estrogen (ER), progesterone (PR), and epidermal growth factor (EGF) receptors (HER2). The cell viability of HER2-overexpressing (SKBR3) and triple-positive (BT474) breast cancer cell lines [but not of a triple-negative cell line (BT20)] was reduced following rfhSP-D treatment at 24 h. Upregulation of p21/p27 cell cycle inhibitors and p53 phosphorylation (Ser15) in rfhSP-D-treated BT474 and SKBR3 cell lines signified G2/M cell cycle arrest. Cleaved caspases 9 and 3 were detected in rfhSP-D-treated BT474 and SKBR3 cells, suggesting an involvement of the intrinsic apoptosis pathway. However, rfhSP-D-induced apoptosis was nullified in the presence of hyaluronic acid (HA) whose increased level in breast tumor microenvironment is associated with malignant tumor progression and invasion. rfhSP-D bound to solid-phase HA and promoted tumor cell proliferation. rfhSP-D-treated SKBR3 cells in the presence of HA showed decreased transcriptional levels of p53 when compared to cells treated with rfhSP-D only. Thus, HA appears to negate the anti-tumorigenic properties of rfhSP-D against HER2-overexpressing and triple-positive breast cancer cells.

## Introduction

The immune surveillance of transformed cells by innate and adaptive immunity remains one of the targeted areas of research for developing therapeutic strategies (1, 2). *In vivo* and *in vitro* studies using cancer models have demonstrated compelling involvement of effector immune cells, soluble factors, and signaling pathways in anti-tumor immune responses. However, the immune system can also aid in the progression of transformed cells by triggering immunosuppression and promoting angiogenesis and metastasis of tumor cells (3, 4).

Human surfactant protein D (SP-D) is a potent innate immune molecule found at pulmonary and non-pulmonary mucosal surfaces (5). It is a member of the collectin family that is involved in the clearance of pathogens and apoptotic/necrotic cells and in the modulation of inflammatory responses (6). SP-D is composed of an N-terminal cysteine-rich domain, a triple-helical collagenous region, an α-helical coiled neck region, and a C-terminal C-type lectin or carbohydrate recognition domain (CRD) (7). The trimeric CRDs recognize carbohydrate or charged patterns on pathogens and allergens, while the collagen region is involved in interactions with receptor molecules present on immune cells in order to trigger clearance mechanisms such as agglutination, enhanced phagocytosis, and oxidative burst (6). SP-D is primarily synthesized and secreted into the airspace of the lungs by alveolar type II and Clara cells, with a key role in surfactant homeostasis by reducing surface tension (6). However, its extrapulmonary existence is well-established now, ranging from the mucosa of the gastrointestinal and reproductive tracts (including ovaries) and nasal cavity to the brain and in various exocrine ducts (8, 9), conjunctiva, cornea, lacrimal gland, nasolacrimal ducts (8), and synovial fluid (10). Protective effects of SP-D against a range of pathogens (6, 11) and allergens (12–16) are well-documented in the literature. However, recent studies have raised the possibility that SP-D may have an important defense role against tumor.

A direct interaction of SP-D with a number of cancer cells (leukemia, lung, prostate, and pancreatic) has been reported to result in the suppression of cancer progression, migration, and invasion, as well as enhanced apoptosis (17–21). The rfhSP-D-treated acute myeloid leukemia (AML) cells were shown to result in cell cycle arrest via activation of G2/M checkpoints, with an increased level of p21 and Try15 phosphorylation of cdc2. rfhSP-D treatment in AML cells also caused activation of pro-apoptotic markers, such as cleaved caspase 9 and downregulation of pro-survival protein HMGA1 (21, 22). Exogenous SP-D treatment has been shown to downregulate epidermal growth factor (EGF) signaling by preventing the binding of EGF to the EGF receptor (EGFR), hence suppressing the cell proliferation, invasion, and migration of A549 human lung adenocarcinoma cells (23). Recently, rfhSP-D has been shown to induce apoptosis in p53 mutant (mt) and wild-type (wt) pancreatic adenocarcinoma (PDAC) cell lines (Panc-1p53 mt, MiaPaCa-2p53 mt, and Capan-2p53wt), via the TNF/Fas-mediated extrinsic pathway (17). Furthermore, rfhSP-D can also suppress epithelial–mesenchymal transition (EMT) and related gene signatures (Vimentin, Zeb1, and Snail) and cell invasiveness in Panc-1 and MiaPaCa-2 cells via downregulation of TGF-β (24). In an ovarian cell line, SKOV3, rfhSP-D again triggered apoptosis via the Fas-mediated pathway (18). In both pancreatic and ovarian cancer cell lines, rfhSP-D treatment caused activation of caspase 3 cleavage and induction of pro-apoptotic genes such as Fas and TNF-α. Furthermore, the mTOR pathway was also affected by rfhSP-D treatment in both ovarian and pancreatic cancer cell lines. rfhSP-D-treated SKOV3 cells show downregulation of Rictor and Raptor mRNA levels, suggesting inhibition of cell proliferation (17, 18). Additionally, the anti-tumor role of rfhSP-D has been reported in androgen-resistant and androgen-responsive prostate cancer cells via p53 and pAkt pathways (19). In a recent bioinformatics study, a higher expression of SP-D in ovarian and lung cancer was found to be associated with a favorable prognosis (20). These studies therefore suggest that SP-D has an immune surveillance function against tumor cells. In this context, this study was aimed at investigating the role of SP-D in breast cancer.

Breast cancer is the most common cancer diagnosed in women worldwide, contributing to almost 60% mortality rate in lower-income countries (25). There is a large variation in the survival rates worldwide, with an estimated 5-year survival rate of 80% in developed countries and below ~40% in low-income countries (26). Designing effective treatments are crucial to improve the survival rates and to ensure the best possible quality of life for cancer survivors. After lung cancer, breast carcinomas are the second most leading cause of mortality (27); ~17.5 million new cases of breast cancer are diagnosed globally, and 8.7 million deaths documented (28). As per GLOBOCAN 2018, ~2 million new cases were identified, and 6.6% of cancer-related deaths were caused by breast cancer. Furthermore, the 5-year prevalence rate of breast cancer was found to be ~6.8 million (25). Breast cancer is characterized by an abnormal growth of malignant cells in the mammary glands. The physiological conditions that lead to breast tumorigenesis include inherited genetic mutation and epigenetic modifications, which can lead to premalignant transformation of mammary cells (29). The development of advanced breast tumor is a consequence of immune selection and immune evasion (30). Breast cancer is subdivided into different molecular subtypes: luminal A, luminal B, triple negative, human EGF receptor (HER)2-enriched, basal, and normal-like tumors (31, 32). The development and metastasis of cancer, including breast cancer, appear to be influenced by innate immune surveillance molecules and associated inflammatory mediators in the tumor microenvironment (33, 34).

Here, we examined possible protective effects of rfhSP-D in triple-negative (ER^−^/PR^−^/HER2^−^), triple-positive (ER^+^/PR^+^/HER2^+^), and HER2^+^-overexpressing breast cancer cell lines. We also examined a possible interaction between SP-D and hyaluronic acid (HA; polymeric nonsulfated glycosaminoglycan), the most abundant component of the extracellular matrix (ECM), which plays an important role in inflammation, angiogenesis, fibrosis, and cancer progression (35). The interaction of breast cancer cells with HA can sustain tumor growth and promote malignant progression. The altered synthesis of HA in early and late stages of ductal breast carcinoma *in situ* (DCIS) microenvironment is correlated with tumor stage progression and invasion (36). Several studies provide strong evidence that HA participates in the regulation of breast tumor cell migration and invasion *in vitro* and tumor growth and progression *in vivo* (37–39). We report, for the first time, that rfhSP-D interacts with HA that negates its anti-tumor properties; i.e., in the presence of HA, rfhSP-D is unable to induce apoptosis in triple-positive and HER2-overexpressing breast cancer cell lines. Therefore, neutralizing the negative effect of HA on rfhSP-D can have crucial implications for the development of therapeutic strategies.

## Materials and Methods

### Expression and Purification of rfhSP-D

Expression and purification of rfhSP-D were performed as published earlier (19). Briefly, *Escherichia coli* BL21 (λDE3) pLysS bacterial strain (Invitrogen) was transformed with plasmid pUK-D1, composed of cDNA sequences for α-helical neck and CRD region of human SP-D. Twenty-five milliliters of bacterial primary inoculum was inoculated into 500 ml of Luria-Bertani medium containing 100 μg/ml ampicillin and 34 μg/ml chloramphenicol (Sigma-Aldrich) and grown to an OD600 of 0.6. The bacterial cells were then induced with 0.5 mM isopropyl β-d-1-thiogalactopyranoside (IPTG) (Sigma-Aldrich) for 3 h. The IPTG-induced bacterial cell pellet was treated with lysis buffer (50 mM Tris–HCl pH 7.5, 200 mM NaCl, 5 mM EDTA pH 8, 0.1% v/v Triton X-100, 0.1 mM phenyl-methyl-sulfonyl fluoride, 50 μg/ml lysozyme) and subsequently sonicated (five cycles, 30 s each). The sonicated was centrifuged at 12,000 × g for 30 min, followed by denaturation and renaturation of rfhSP-D inclusion bodies using refolding buffer (50 mM Tris–HCl pH 7.5, 100 mM NaCl, 10 mM 2-mercaptoethanol) containing 8 M urea. The dialysate was then loaded onto a maltose–agarose affinity column (5 ml; Sigma-Aldrich), and rfhSP-D was eluted using 10 mM EDTA buffer containing 50 mM Tris–HCl pH 7.5 and 100 mM NaCl. Eluted rfhSP-D fractions were then tested for endotoxin levels using a QCL-1000 Limulus amebocyte lysate system (Lonza). The endotoxin levels were found to be ~5 pg/μg of rfhSP-D. The purity of protein was analyzed via 15% w/v SDS-PAGE, and its immunoreactivity was assessed via western blotting.

### Cell Culture and Treatments

Human breast cancer cell lines, triple-negative BT20 (ER^−^/PR^−^/HER2^−^) (ATCC-HTB19), triple-positive BT474 (ER^+^/PR^+^/HER2^+^) (ATCC-HTB20), and HER2-positive SKBR3 (ER^−^/PR^−^/HER2^+^) (ATCC-HTB30), were cultured in complete RPMI medium (Sigma-Aldrich), supplemented with 10% v/v fetal bovine serum (FBS), 2 mM l-glutamine, 100 U/ml penicillin (Sigma-Aldrich), 100 μg/ml streptomycin (Sigma-Aldrich), and 1 mM sodium pyruvate (Sigma-Aldrich) and left to grow at 37°C under 5% v/v CO_2_. Since these cell lines were adherent, cells were detached using 2× trypsin–EDTA (0.5%) (Fisher Scientific) for 10 min at 37°C, then centrifuged at 1,500 rpm for 5 min, and resuspended in complete RPMI. Cell number and viability were assessed by mixing an equal volume of the cell suspension and Trypan Blue (0.4% w/v) (Fisher Scientific) and by counting using a hemocytometer with a Neubauer chamber (Sigma-Aldrich).

### Fluorescence Microscopy

BT20, BT474, and SKBR3 (50,000) cells were grown on coverslips and incubated with rfhSP-D (10 μg/ml) in serum-free RPMI medium for 1 h for cell binding analysis and 24 h for apoptosis induction. For binding experiments, the coverslips were washed three times with phosphate-buffered saline (PBS) and incubated with polyclonal anti-rabbit human SP-D antibody (1:200) (Medical Research Council Immunochemistry Unit, Oxford) for 1 h at room temperature (RT). Following washes with PBS, the cells were incubated with goat anti-rabbit IgG conjugated to Alexa Fluor® 488 (1:200) (Abcam) and Hoechst (1:10,000) (Sigma-Aldrich) for immunofluorescence analysis. For apoptosis induction analysis, the cells were incubated with FITC Annexin V (1:200) and propidium iodide (PI) (1:200) diluted in Annexin V-binding buffer for 15 min at RT in dark. After washing with PBS three times, the coverslips were mounted on slides and viewed under an HF14 Leica DM4000 microscope.

### Flow Cytometry

BT20, BT474, and SKBR3 (0.4 × 10^6^) cells were grown in a six-well-plate and incubated with rfhSP-D (20 μg/ml), along with an untreated control, for 24 h. The cells were then detached using 2 × trypsin–EDTA (0.5%) (Fisher Scientific) and centrifuged at 1,500 × *g* for 5 min. Staurosporine (1 μM/ml; Sigma-Aldrich) was used as a positive control of apoptosis. Following washes with PBS, the cells were incubated for 15 min with goat anti-rabbit IgG conjugated to Alexa Fluor® 488 (1:200) (Abcam) and Hoechst (1:10,000) (Sigma-Aldrich) for apoptosis analysis. After washing with PBS three times, apoptosis was measured using a NovoCyte flow cytometer. Compensation parameters were acquired using unstained, untreated FITC-stained, and untreated PI-stained cells for all three cell lines. For solid-phase studies, six-well-plates were coated with HA (20 μg/ml) (a kind gift from Prof. Ivan Donati, Department of Life Sciences, University of Trieste) overnight at 4°C with and without rfhSP-D (20 μg/ml). The wells were washed with PBS, and 0.4 × 10^6^ cells were added to the HA/rfhSP-D-coated wells and incubated 37°C for 24 h. For proliferative studies, the cells were washed with PBS and incubated with anti-mouse Ki-67 (BioLegend) diluted in a permeabilization reagent of the FIX&PERM kit (Fisher Scientific) for 30 min at RT. After PBS washes, the cells were probed with a goat anti-mouse-FITC conjugate (1:200) (Fisher Scientific) for 30 min at RT in the dark. Cells (12,000) were acquired for each experiment and compensated before plotting the acquired data.

### Purification of Full-Length SP-D From Breast Cancer Cell Lines

BT20, BT474, and SKBR3 (0.4 × 10^6^) culture media were collected and centrifuged at 5,000 rpm for 10 min. The supernatants were passed through a maltose–agarose affinity column. The bound full-length SP-D was eluted using an elution buffer, containing 50 mM Tris–HCl pH 7.5, 100 mM NaCl, and 10 mM EDTA. Full-length SP-D protein yield of 1 μg/ml was obtained from 100 ml of culture medium. The purity of eluted fractions (10 μg/ml) was analyzed via 15% w/v SDS-PAGE, and its immunoreactivity was determined via western blotting. For western blotting analysis, the eluted full-length SP-D was heated for 10 min at 100°C and subjected to SDS-PAGE (12% v/v). The proteins were then electrophoretically transferred onto a nitrocellulose membrane (320 mA for 2 h) in 1× transfer buffer (25 mM Tris–HCl pH 7.5, 190 mM glycine, and 20% methanol). The membrane was blocked using 5% w/v dried milk powder (Sigma-Aldrich) in PBS overnight at 4°C, followed by washing with PBS three times for 5 min each. The membrane was then incubated with polyclonal anti-rabbit human SP-D antibody (MRC Immunohistochemistry Unit, Oxford) (1:1,000), followed by probing with protein A–HRP (Sigma-Aldrich) (1:1,000). Following washing with PBST, the membrane was developed using a 3′-diaminobenzidine (DAB) substrate kit.

### Apoptosis Analysis of Breast Cancer Cells Treated With BT474-Derived Full-Length SP-D

The purified full-length SP-D from breast cancer culture was subjected to apoptosis assay using an Annexin V/FITC kit (BioLegend). BT20, BT474, and SKBR3 cells (0.4 × 10^6^) were incubated with 20 μg/ml of culture-purified SP-D and incubated for 24 h. The cells were then detached using 2 × trypsin–EDTA (0.5%) (Fisher Scientific) and centrifuged at 1,500 × g for 5 min. Following washes, the cells were then incubated with both FITC and PI dye (1:200) for 15 min in the dark. After washing with PBS, the cells were subjected to flow cytometric analysis. Compensation parameters were acquired using unstained, untreated FITC-stained, and untreated PI-stained cells for all three cell lines.

### Immunohistochemical Analysis

Surgical breast cancer tissues and adjacent peritumoral mammary parenchymas were selected following ethical approval by the University Hospital of Palermo Ethical Review Board (approval number 09/2018). TNBC, HER2+, luminal B, and luminal A breast cancer tissues were used, while normal breast tissue was used as a control. Immunohistochemistry (IHC) was performed using a polymer detection method. Briefly, tissue samples were fixed in 10% v/v buffered formalin and paraffin embedded. Four-micrometer-thick tissue sections were deparaffinized and rehydrated. The antigen unmasking technique was carried out using Novocastra Epitope Retrieval Solutions, pH 6 EDTA based (Leica Biosystems), in a thermostatic bath at 98°C for 30 min. Sections were brought to RT and washed in PBS. After neutralization of the endogenous peroxidase with 3% v/v H_2_O_2_ and Fc blocking (Novocastra, Leica Biosystems), the samples were incubated overnight at 4°C with mouse anti-human SP-D monoclonal antibody (1:800) (Abcam). Staining was revealed via a polymer detection kit (Novocastra, Leica Biosystems) and 3-amino-9-ethylcarbazole (AEC, Dako, Denmark) substrate chromogen. Slides were counterstained with Harris hematoxylin (Novocastra, Leica Biosystems). Alcian blue staining was carried out to detect the presence of mucins in breast cancer tissue sections, according to the manufacturer's kit Bioptica (04-160802). Slides were analyzed under the Axio Scope A1 optical microscope (Zeiss) and microphotographs were collected through the Axiocam 503 color digital camera (Zeiss) using the Zen 2 software.

### Adhesion Assay

BT20, BT474, and SKBR3 (0.5 × 10^5^) cells were labeled with FAST DiI fluorescent dye (Molecular Probes, Invitrogen) and allowed to adhere to 96 microtiter wells pre-coated with 20 μg/ml of HA, rfhSP-D, and bovine serum albumin (BSA). The adhesion of cells was measured after 35 min of incubation at 37°C under 5% CO_2_. The non-adhered cells were washed off with PBS, and the remaining cells were lysed using 10 mM Tris–HCl pH 7.4 + 0.1% v/v SDS. The plate was read at 544 nm using Infinite 200 (Tecan). Results were expressed as adhesion percentage with reference to a standard curve established using an increasing number of FAST DiI-labeled cells.

### Intracellular Signaling Analysis

Signaling pathway was analyzed using the PathScan Intracellular Signaling Array Kit (Cell Signaling Technology). Briefly, 0.5 × 10^6^ breast cancer cell lines were grown in a six-well-plate in serum-free RPMI medium. Cells were then left to adhere to HA and rfhSP-D (20 μg/ml) pre-coated plates and incubated at 37°C for 25 min. The unbound cells were then washed with cold PBS and lysed in ice-cold cell lysis buffer containing a cocktail of protease inhibitors (Roche Diagnostics). The Array Diluent Blocking buffer was added to each well on the multi-well gasket at RT for 15 min. After decanting the Array Blocking Buffer, 75 μl of the total lysate (0.8 mg/ml) was added to each well and incubated for 2 h at RT on an orbital shaker. The well contents were decanted and washed with 100 μl of 1× Array Wash Buffer three times (5 min each wash). The wells were then incubated with biotinylated detection cocktail antibody for 1 h at RT, followed by incubation with streptavidin-conjugated DyLight 680 for 30 min. The fluorescence readout was measured via LI-COR Biosciences Infrared Odyssey imaging system (Millennium Science), and data were processed by the software Image Studio 5.0.

### Western Blotting

BT20, BT474, and SKBR3 (0.4 × 10^6^) cells were seeded in a six-well-plate and treated with rfhSP-D (20 μg/ml) for 24 h, along with an untreated control, in a serum-free RPMI medium. After removing the medium, the cells were lysed using a lysis buffer (50 mM Tris–HCl pH 6.8, 2% v/v SDS, 2% v/v β-mercaptoethanol, 10% v/v glycerol and 0.1% w/v bromophenol blue). The lysed cells were then sonicated for 15 s, and the sonicated samples were heated for 10 min at 100°C and subjected to SDS-PAGE (12% v/v). The proteins were then electrophoretically transferred onto a nitrocellulose membrane (320 mA for 2 h) in 1× transfer buffer (25 mM Tris–HCl pH 7.5, 190 mM glycine, and 20% v/v methanol). The membrane was blocked using 5% w/v dried milk powder (Sigma-Aldrich) in PBS overnight at 4°C, followed by washing with PBS three times (5 min each). For apoptosis studies, the membrane was incubated with rabbit anti-human caspase primary antibodies (anti-cleaved caspase 9 and anti-cleaved caspase 3; Cell Signaling) at RT for 1 h. The membrane was washed with PBST (PBS + 0.05% Tween 20) three times, 10 min each, followed by incubation with secondary goat anti-rabbit IgG horseradish peroxidase (HRP) conjugate (1:1,000; Fisher Scientific) for 1 h at RT. Following washes with PBST, the membrane was developed using a DAB substrate kit (Thermo Fisher).

### ELISA

For HA (1,500 kDa) binding analysis, a constant concentration of HA (20 μg/ml) was coated overnight at 4°C using carbonate/bicarbonate (CBC) pH 9.6 buffer, followed by blocking with 2% w/v BSA at 37°C. Recombinant maltose binding protein (MBP) was used as a negative control in this experiment. After three washes with PBST, rfhSP-D (5, 10, or 20 μg/ml) was incubated in buffer containing 5 mM CaCl_2_ for 2 h at 37°C. After washing with PBST three times, the binding was assessed by polyclonal anti-rabbit human SP-D antibody (1:5,000) and incubated at 37°C for 1 h. The wells were washed again with PBST three times and incubated with protein A–HRP secondary conjugate (1:5,000) (Sigma-Aldrich) for 1 h at 37°C. After washes with PBST, the binding was detected using 3,3′,5,5′-tetramethylbenzidine (TMB) substrate (Sigma-Aldrich). The reaction was stopped using 2 N H_2_SO_4_, and the absorbance was read at 450 nm using an iMark™ microplate absorbance reader (Bio-Rad).

### Quantitative RT-PCR

BT20, BT474, and SKBR3 (0.4 × 10^6^) cells were added to six-well-plate pre-coated with HA (20 μg/ml), HA + rfhSP-D (20 μg/ml), and rfhSP-D (20 μg/ml) and incubated at 37°C for various time points. The cell pellet for each time point was subjected to RNA extraction using GenElute Mammalian Total RNA Purification Kit (Sigma-Aldrich). RNA samples were then treated with DNase I (Sigma-Aldrich), and the total RNA concentration and purity were determined using a 260:280 nm ratio using NanoDrop 2000/2000c (Thermo Fisher Scientific). Two micrograms of total RNA was used for cDNA synthesis using High Capacity RNA to cDNA kit (Applied Biosystems). Primer sequences used in this study were designed for specificity using the Primer-BLAST software (Basic Local Alignment Search Tool) (http://blast.ncbi.nlm.nih.gov/Blast.cgi). p21 and p27 mRNA levels were estimated by quantitative RT-PCR (qRT-PCR) using the following primers: 5′-TGGAGACTCTCAGGGTCGAAA-3′ and 5′-CGGCGTTTGGAGTGGTAGAA-3′ for p21, and 5′-CCGGTGGACCACGAAGAGT-3′ and 5′-GCTCGCCTCTTCCATGTCTC-3′ for p27. Briefly, the qPCR reaction consisted of 5 μl of iQ SYBR Green Supermix (Bio-Rad, Milan, Italy), 75 nM of forward and reverse primers, and 500 ng template cDNA in a 10 μl final reaction volume. qRT-PCR was performed on a Rotor-Gene 6000 (Corbett, Explera, Ancona, Italy). The melting curve of the reactions was recorded between 55 and 99°C with a hold every 2 s. The comparative quantification (CQ) method was used to determine the relative amount of gene expression in each sample, and the data analyzed using Rotor-Gene 1.7 software (Corbett Research) (40). The CQ method is specific in calculating the efficiencies of each gene for each individual PCR and is based on the second differential maximum method. The samples were normalized using the expression of human TATA binding protein (TBP) rRNA. Assays were conducted in triplicates.

### Statistical Analysis

Graphs were generated using GraphPad Prism 6.0; the statistical analysis was carried out using an unpaired one-way ANOVA test. Error bars represent SD or SEM (*n* = 3), as indicated in the figure legends. The significant values were measured based on ^*^p < 0.05, ^**^p < 0.01, ^***^p < 0.001, and ^****^p < 0.0001 between treated and untreated samples.

## Results

### A Recombinant Form of Truncated Human SP-D Binds Breast Cancer Cell Lines

**Figure 1:**
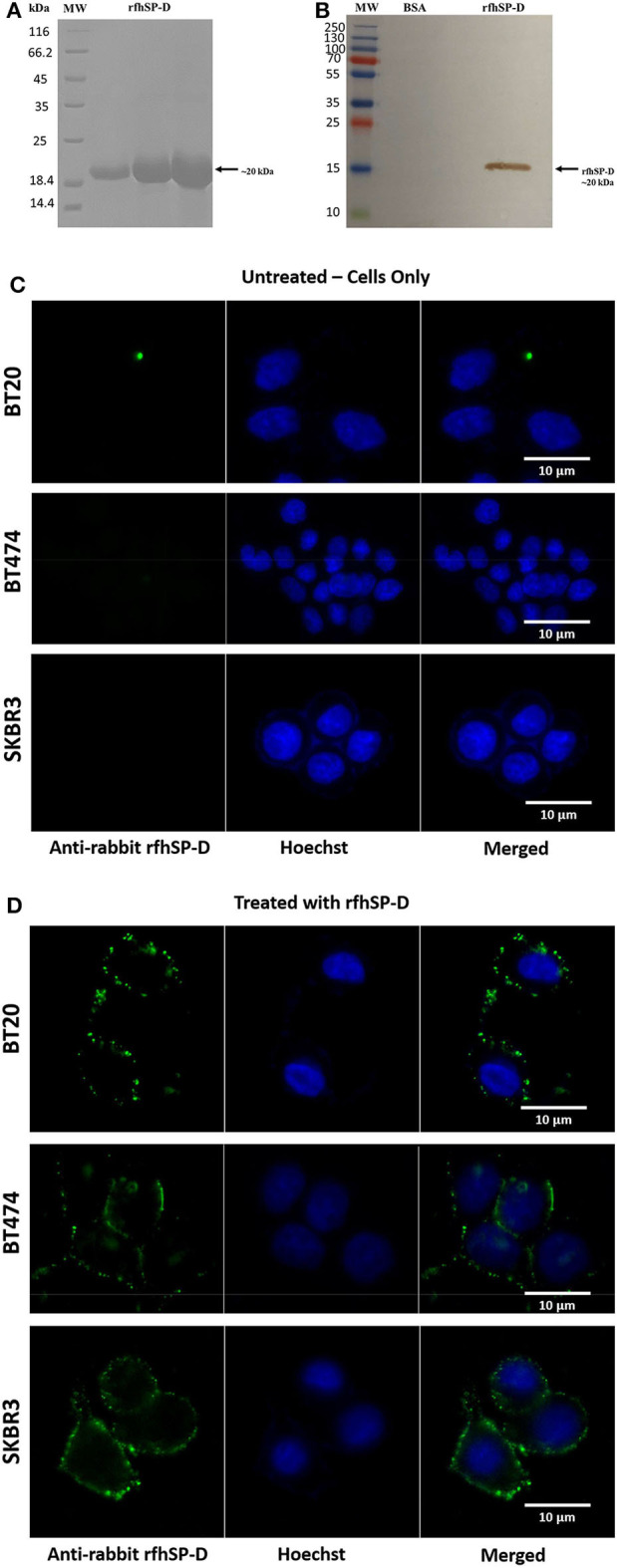
SDS-PAGE **(A)** and immunoblot profile **(B)** of the purified recombinant form of human surfactant protein D (rfhSP-D). Expression of rfhSP-D under the bacteriophage T7 promoter, expressed as ~20 kDa insoluble protein. Eluted fractions were passed through a maltose–agarose column, which appeared as a single band at ~20 kDa. Immunoreactivity of affinity-purified rfhSP-D was examined via western blotting; lane 1: BSA as a negative control; lane 2: purified rfhSP-D (5 μg/ml). Binding of rfhSP-D (10 μg/ml) to breast cancer cell lines using fluorescence microscopy, following 1 h incubation at 4°C **(C,D)**. The nucleus of the cells was stained with Hoechst (1:10,000), and both untreated (cells only) **(C)** and rfhSP-D-treated **(D)** cells were probed with polyclonal anti-human SP-D/FITC antibody (1:200). Membrane localization of the bound proteins was only detected in the treated cells, while no FITC was detected in the untreated control (cells only).

rfhSP-D comprising the eight Gly-X-Y repeats and the neck and CRD regions of human SP-D was affinity-purified and migrated at ~20 kDa on SDS-PAGE under reducing conditions (Figure 1A). The immunoreactivity of purified rfhSP-D was confirmed via western blotting using polyclonal anti-human SP-D antibody raised in rabbit against native human SP-D purified from lung lavage (Figure 1B). BSA was used as a negative control protein. Purified rfhSP-D (5 μg/ml) bound breast cancer cell lines, triple-negative BT20 (ER^−^/PR^−^/HER2^−^), triple-positive BT474 (ER^+^/PR^+^/HER2^+^), and HER2-positive SKBR3 (ER^−^/PR^−^/HER2^+^) (Figure 1D). The nucleus of the cell was stained with Hoechst dye, while the membrane localization of the bound rfhSP-D was revealed using green FITC/anti-human SP-D conjugate. All rfhSP-D-treated breast cancer cell lines showed a similar “cluster”-like binding pattern on the cell membrane (Figure 1D). No FITC was detected in the untreated controls (Figure 1C), probed with both primary and secondary antibodies, suggesting the specificity of rfhSP-D binding.

### Apoptosis Induction by rfhSP-D in Breast Cancer Cell Lines

**Figure 2:**
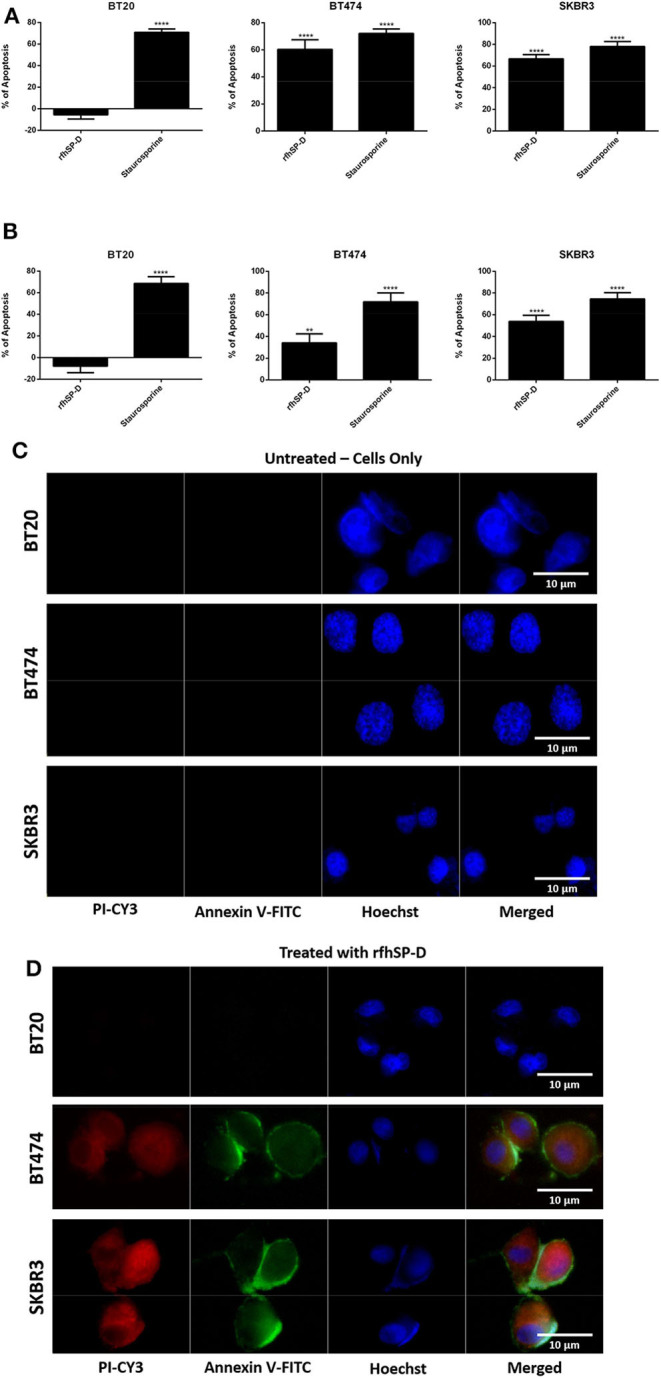
Flow cytometric analysis of apoptosis induction in breast cancer cells treated with 20 μg/ml of immobilized **(A)** and solution-phase **(B)** rfhSP-D for 24 h. For both Annexin V/FITC and DNA/PI staining, 12,000 cells were acquired and plotted. A significant difference was seen among treated and untreated (cells only) samples, as evident by the shift in the fluorescence intensity. The data were expressed as the mean of three independent experiments (*n* = 3) done in triplicates ± SEM. Statistical significance was established using the unpaired one-way ANOVA test (**p < 0.01 and ****p < 0.0001). The percentage of apoptosis was calculated by normalizing the treated cells with their untreated counterparts. Fluorescence microscopy analysis of apoptosis induction in breast cancer cells treated with rfhSP-D (20 μg/ml) for 24 h, using an Annexin V with a propidium iodide (PI) staining kit; untreated (cells only) controls **(C)** and rfhSP-D-treated cells **(D)**. The nucleus was stained with Hoechst (1:10,000), and the cell membrane was stained positively with Annexin V and PI (1:200) in treated cell lines, suggesting that cells treated with rfhSP-D induced apoptosis at 24 h, where translocation of PS to the outer plasma membrane was able to bind Annexin V due to loss of membrane integrity and PI stain was taken, which stained the DNA of apoptotic cells. No Annexin V/PI staining was detected in untreated cells.

The quantitative and qualitative analyses of apoptosis induction by rfhSP-D were performed using flow cytometry (Figures 2A,B) and immunofluorescence microscopy (Figures 2C,D). A significant proportion of BT474 and SKBR3 breast cancer cells treated with rfhSP-D (20 μg/ml) underwent apoptosis at 24 h as revealed by FACS analysis (Table 1). BT474 (~61%) and SKBR3 (~68%) cell lines were more susceptible to apoptosis induction when immobilized rfhSP-D (20 μg/ml) (Figure 2A) was used; there was no effect on the BT20 cell line. rfhSP-D in solution also induced apoptosis in BT474 (~34%) and SKBR3 (~53%) (Figure 2B); again, rfhSP-D had no significant effect on the BT20 cell line (Figure 2A, Table 1). The integrity of the cell membrane was intact in untreated cells, and hence, the cells were still viable, blocking the translocation of phosphatidylserine (PS) from the inner cell membrane to outer plasma membrane and preventing the PS–Annexin V interaction. Nearly 56% BT474 and SKBR3 cells were both stained positive for FITC and PI, suggesting that annexin V/FITC was able to bind to PS found on the cell surface of the cells undergoing apoptosis. However, there were more BT474 cells that stained for PI alone than the SKBR3 cell line, suggesting that BT474 cells were either at the late stage of apoptosis or undergoing necrosis. The percentage of viable cells in the untreated (cells only) sample was significantly higher as compared to the treated sample, BT474 (~92%) and SKBR3 (~96%), suggesting that apoptosis induction was a rfhSP-D protein-specific effect (Figure 2A). Staurosporine (1 μM/ml) was used as a positive control for apoptosis studies. Staurosporine-treated BT20, BT474, and SKBR3 showed ~70% of apoptosis induction at 24 h (Figures 2A,B). Staurosporine is a well-known therapeutic, potent apoptosis inducer, which is suggested to inhibit tumor cell growth and proliferation by triggering cell death via an intrinsic apoptotic pathway (41). Fluorescence microscopy analysis in BT474 and SKBR3 cell lines treated with rfhSP-D revealed a positive staining for cell membrane integrity marker, Annexin V (conjugated to FITC), and disoriented cell membrane morphology (Figures 2C,D). Thus, PI-positive staining was seen only in treated cell lines (Figure 2D) compared to untreated control cells (Figure 2C), indicating no occurrence of late apoptotic or necrosis. In contrast, no positive staining of Annexin V or PI was detected in BT20 treated cells, which were similar to the untreated control.

**Table 1:** Flow cytometric analysis of apoptosis induction in breast cancer cells treated with 20 μg/ml of immobilized and solution-phase rfhSP-D for 24 h.

	**Immobilized phase**			**Solution phase**		
	**Background apoptosis (%)**	**Normalized apoptosis with rfhSP-D (%)**	**Normalized apoptosis with staurosporine (%)**	**Background apoptosis (%)**	**Normalized apoptosis with rfhSP-D (%)**	**Normalized apoptosis with staurosporine (%)**
BT20	5	7	70	5	12	70
BT474	8	61	70	8	34	70
SKBR3	4	68	70	4	53	70

### rfhSP-D Induces Apoptosis in BT474 and SKBR3 Cell Lines via Intrinsic Pathway

**Figure 3:**
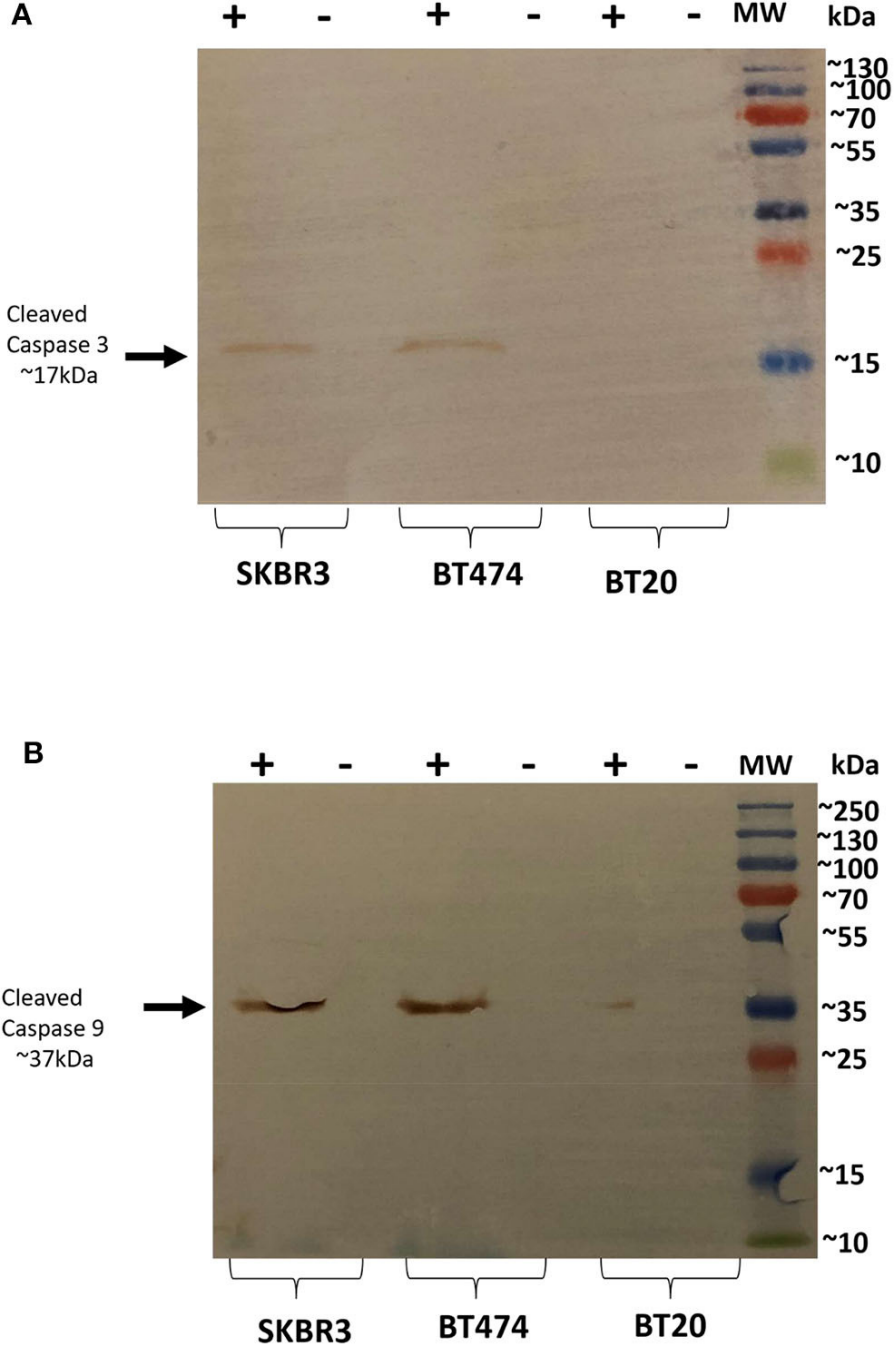
Caspase activation in breast cancer cell lines following rfhSP-D treatment at 24 h. Western blot analysis showed presence of cleaved caspase 3 at 17kDa in the cells treated with rfhSP-D for 24 h. **(A)** Western blot analysis showed presence of cleaved caspase 9 at 37kDa **(B)**, suggesting induction of the intrinsic pathway.

Since rfhSP-D caused apoptosis in BT474 and SKBR3 cells, we further examined the likely apoptotic pathway being triggered following rfhSP-D treatment. Apoptosis can be initiated through intrinsic or extrinsic pathways. Expression of caspases was tested in rfhSP-D-treated breast cancer cell lines. Western blotting analysis of rfhSP-D-treated BT474 and SKBR3 cells at 24 h revealed cleavage of caspase 9 (~37 kDa) and 3 (~17 kDa), suggesting the involvement of the intrinsic apoptosis pathway (Figure 3). As expected, cleavage of caspases 3 (Figure 3A) and 9 (Figure 3B) was not detected in BT20 and untreated (cells only) controls. Furthermore, cleaved caspase 8 was tested as a marker for the extrinsic pathway, but no difference was observed between rfhSP-D-treated cell lines and untreated controls (data not shown).

### Expression of Human SP-D and HA in Breast Cancer Tissues

**Figure 4:**
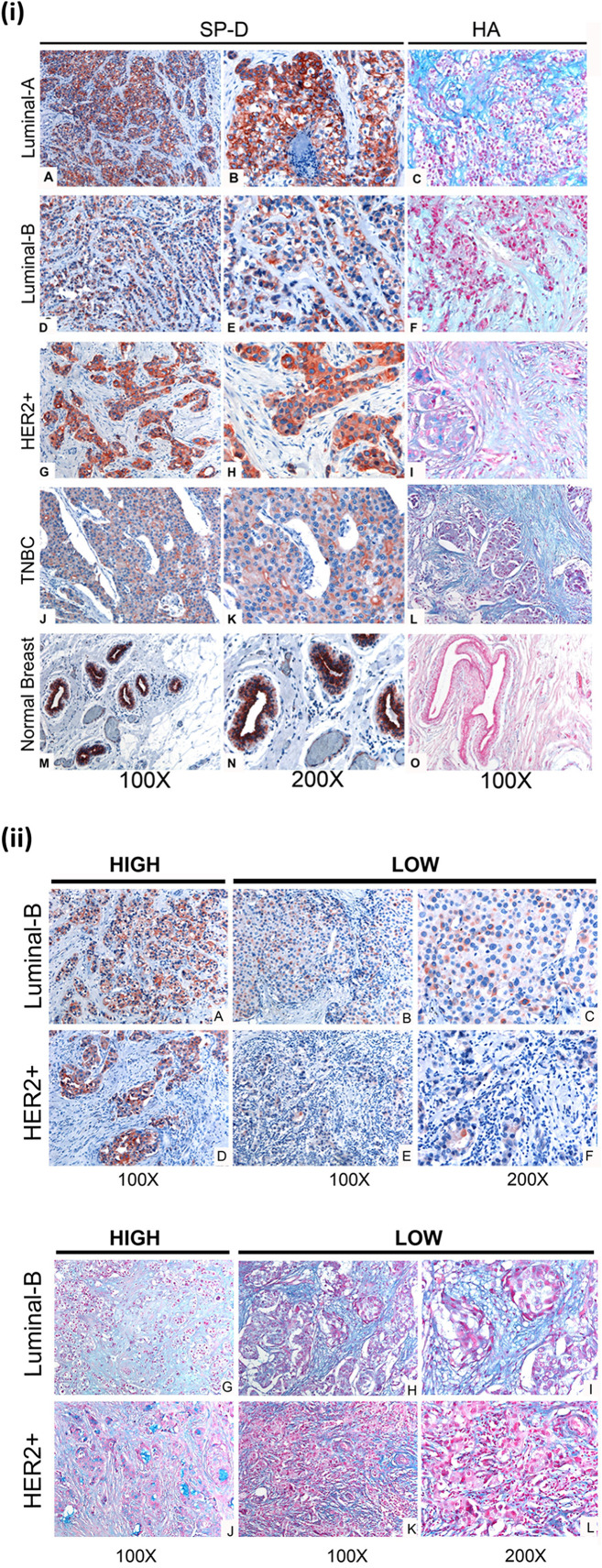
(i) SP-D and Hyaluronic acid (HA) presence in different histotypes of neoplastic breast **(A–L)**, and normal ductal mammary epithelium **(M–O)**. **(A,D,G,J)**. A high variability of SP-D expression between different histotypes is shown, although the positivity of the signal is always attributable to neoplastic cells, as can be observed from the higher- resolution images in the right part of the figure **(B,E,H,K)**. AEC (red) chromogen was used to visualize the binding of anti-human SP-D antibodies. **(C,F,I,L,O)** Histochemical staining with Alcian Blue highlighted HA distribution in breast cancer and normal tissue sections; in particular, the staining was visible in tumor-associated stroma. (ii) Immunohistochemical and histochemical analysis in Luminal-B and Her2+/ER-/PR- breast carcinoma sections of SP-D **(A–F)** and HA **(G–L)** expression, respectively **(B)**. A high variability of SP-D and HA expression within the same isotypes is shown. It is possible to notice a slight inverse correlation between SP-D and HA expression. AEC (red) chromogen was used to visualize the binding of anti-human SP-D antibodies, whereas histochemical staining with Alcian Blue highlighted HA distribution.

The expression of human SP-D was analyzed in eight cases of invasive ductal carcinoma, no special type (NSP), comprising two cases for each molecular class (luminal A, luminal B, Her2-Neu, and triple negative) based on the expression of the estrogen receptor (ER), the progesterone receptor (PR), and the human EGF receptor 2 (Her2-Neu) status. Among biological variables considered, besides the different molecular class, the neoplastic tissues presented a different grade tumor, computed with the Nottingham score, varying from moderate (G2) to undifferentiated (G3). Indeed, the molecular classification allows us to identify four different classes based on the expression of the ER and PR and the human EGF receptor 2 (Her2-Neu) status and called luminal A, luminal B, Her2-Neu, and triple negative, respectively. The molecular subtypes reflect the neoplastic heterogeneity of breast cancer and differ in gene expression patterns, clinical and morphological features, response to treatment, and outcome. Our aim was to evaluate the presence and distribution of SP-D in all molecular subtypes of breast cancer to dissect its biological role in neoplastic progression and to consider a potential predictive and/or prognostic marker of mammary carcinoma. Our data appear to suggest heterogeneous inter-tumor and intra-tumor expressions of SP-D within the molecular subtypes. IHC staining for SP-D highlighted its cytoplasmic expression in both the neoplastic tissue and the healthy peritumoral mammary parenchyma, as previously demonstrated (20). Indeed, the presence of SP-D was evident in cytoplasmic labeling and was highly expressed by the ductal epithelium of peritumoral mammary parenchyma and the neoplastic subclones of luminal A type, while its reduced expression was evident in triple-negative breast cancer (TBNC) (Figure 4A). For the luminal B and HER2 groups, a heterogeneous expression of SP-D was observed (Figures 4i,ii). We also examined serial sections for the localization of HA within the tumor microenvironment following histochemical analysis of normal and breast cancer tissue specimens. As shown in Figure 4i, Alcian blue staining highlighted the presence of acid mucins, containing HA and sialic acid (42–44), in the tumor stroma, around the tumor cells and was very faint in the peritumoral mammary parenchyma in all the molecular classes of breast cancer considered. The luminal B and HER2 specimens were characterized by a variable SP-D expression within the tumor. In the luminal B and HER2 groups, Alcian blue staining also showed a greatly heterogeneous expression of HA. Thus, a stronger staining for Alcian blue was evident where a lower presence of SP-D was detected, suggesting that a modification of ECM might be associated with a variable distribution of SP-D (Figures 4A,G–L).

### rfhSP-D Binds to High-Molecular-Weight HA

**Figure 5:**
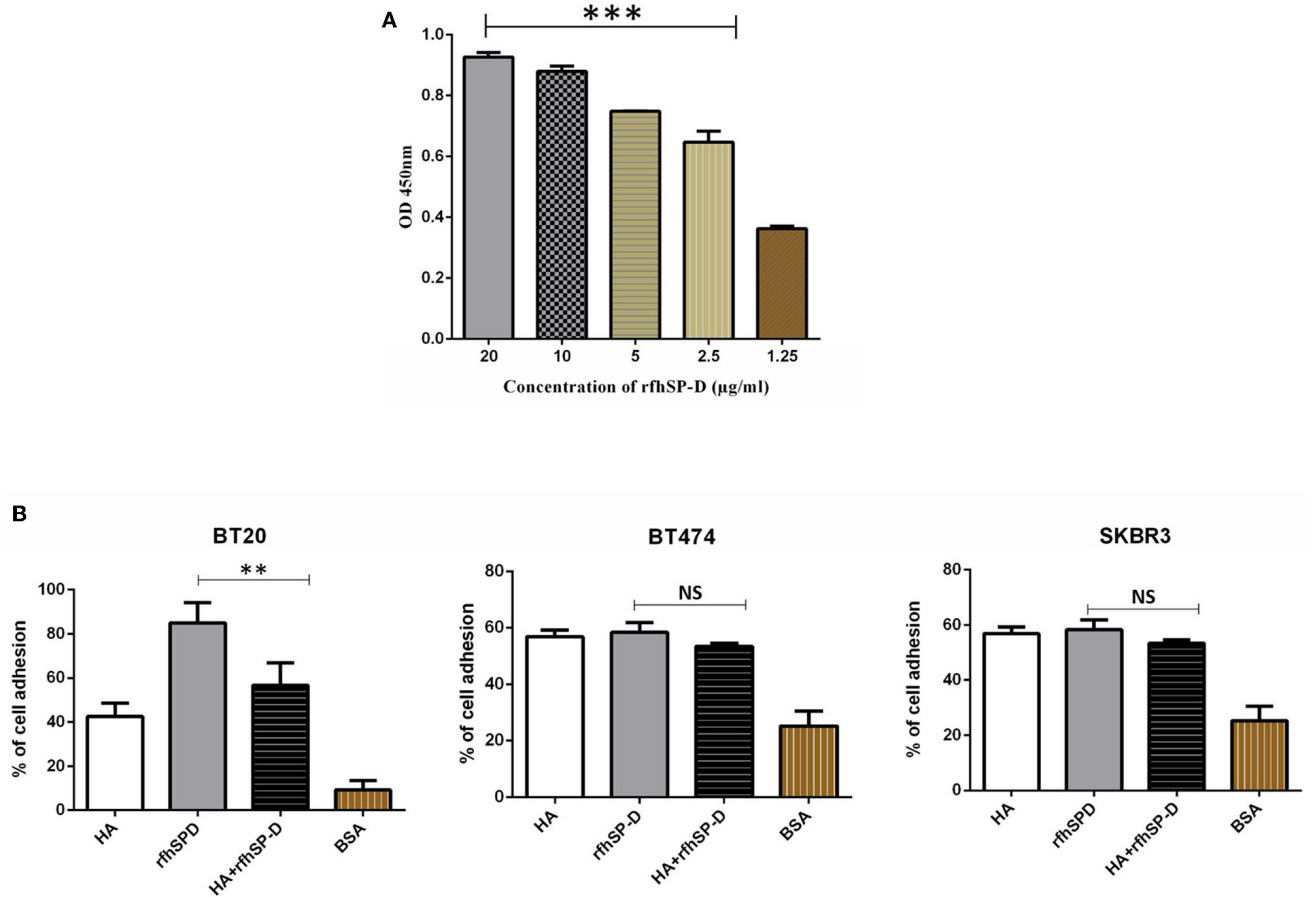
Interaction of rfhSP-D with HA. Binding of varied concentrations of rfhSP-D to HA (20 μg/ml) by ELISA **(A)**. The effects of rfhSP-D on Breast cancer cell lines adhesion **(B)**. Breast cancer cells were labeled with the FAST DiI fluorescent dye and allowed to adhere to 96 microtiter wells pre-coated with HA, rfhSP-D, and BSA. The data is expressed as the mean of three independent experiments. The adhesion of cells was measured after 35 minutes of incubation at 37°C under 5% CO_2_. Results are expressed as adhesion percentage with reference to a standard curve established using an increasing number of FAST DiI -labeled cells. The data is expressed as the mean of three independent experiments (n = 3) done in triplicates ± SEM. Statistical significance was established using the unpaired one-way ANOVA test (**p < 0.01 and ***p < 0.001). The statistical analysis was performed between rfhSP-D and HA + rfhSP-D -treated breast cancer cells.

Confirmation of the presence of HA in the breast cancer tissues prompted us to examine if rfhSP-D binds to HA and can modulate the nature of rfhSP-D–breast cancer cell interaction. In direct binding ELISA, rfhSP-D bound to solid-phase high-molecular-weight (1,500 kDa) HA in a dose-dependent manner (Figure 5A). Immobilized rfhSP-D (20 μg/ml) alone as well as HA (20 μg/ml)-bound rfhSP-D were then allowed to interact with breast cancer cell lines. Cell adhesion assay was performed by labeling the cells with the fluorescent probe FAST DiI (Figure 5B). All three breast cancer cell lines (BT20, BT474, and SKBR3) were able to adhere to HA, to rfhSP-D, and to HA-bound rfhSP-D. rfhSP-D enticed a greater cell adhesion when compared to HA alone or HA-bound rfhSP-D; all three cell lines bound to similar extent (Figure 5B).

### HA Binding Modulates the Ability of rfhSP-D to Induce Apoptosis

**Figure 6:**
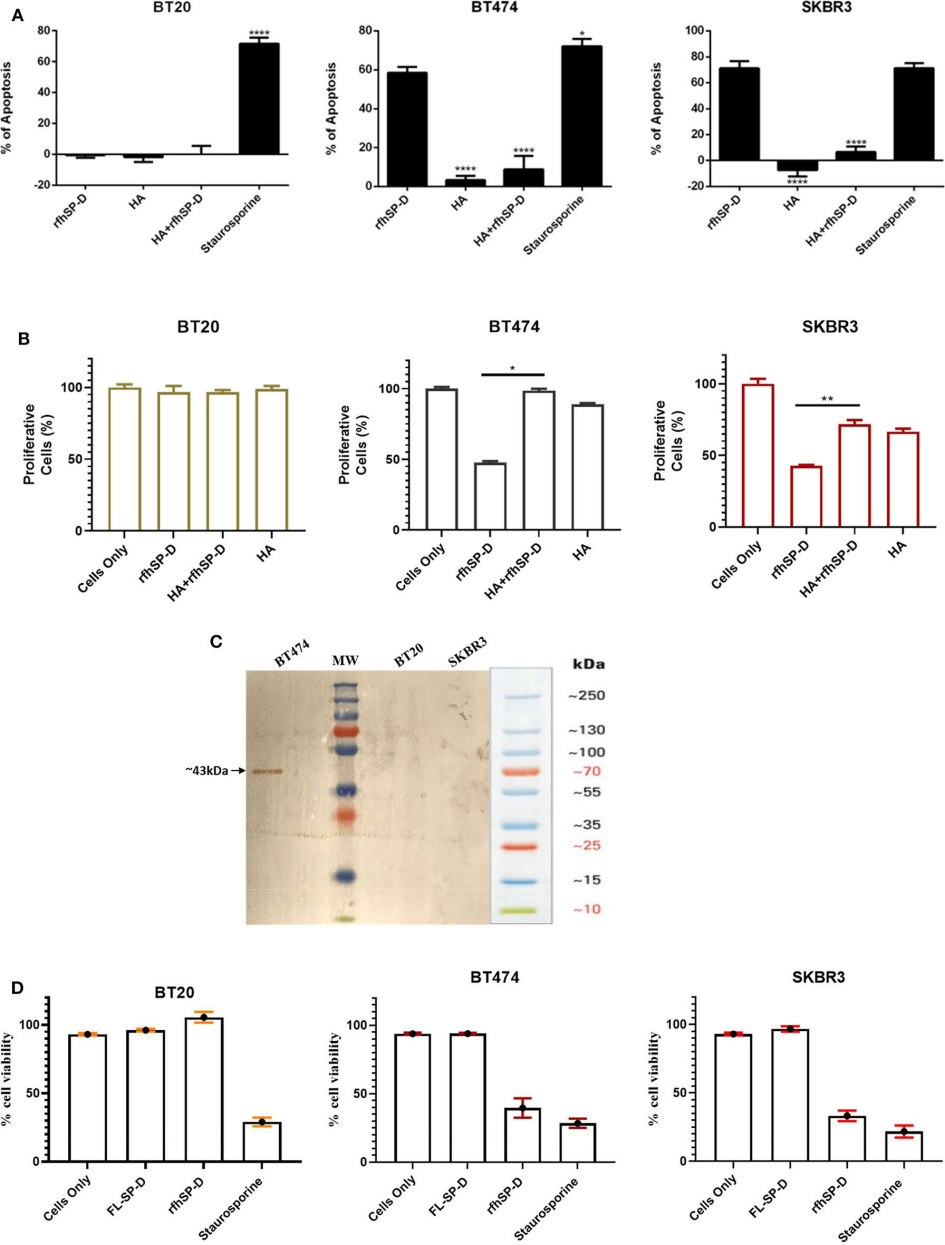
Apoptosis induction in BT20, BT474, and SKBR3 cell lines following HA challenge in the presence and absence of rfhSP-D **(A)**. The data were expressed as the mean of three independent experiments (*n* = 3). A significant difference was seen among treated and untreated samples, as made evident by the shift in the fluorescence intensity. Staurosporine (1 μM/ml) was used as a positive control. Proliferative effects of rfhSP-D treatment on BT474 and SKBR3 breast cancer cell lines **(B)**. BT474 and SKBR3 cells were seeded in wells pre-coated with HA, HA + rfhSP-D, and rfhSP-D alone. The percentage of proliferative cells was evaluated by staining with mouse anti-human KI-67 antibody, and KI-67-stained cells were measured via flow cytometry. The data were generated from at least three independent experiments (*n* = 3) and presented as mean ± SD (*p < 0.1, **p < 0.01, and ****p < 0.0001). The statistical analysis was performed between rfhSP-D and HA + rfhSP-D-treated breast cancer cells. The secretion levels of FL-SP-D were confirmed and analyzed via western blotting **(C)**. Culture medium collected from BT20, BT474, and SKBR3 cell lines was passed through a maltose Agarose column, and the eluted fractions were validated via western blotting; FL-SP-D was detected at ~43 kDa only for BT474. Both BT20 and SKBR3 cell lines did not secrete any FL-SP-D. Secreted FL-SP-D by BT474 was tested for its ability to induce apoptosis **(D)**. No effect of secreted FL-SP-D was seen in terms of cell viability and apoptosis induction.

The implication of HA–rfhSP-D interaction on apoptosis induction in breast cancer cells was investigated (Figure 6A). Breast cancer cells incubated with pre-coated HA, HA + rfhSP-D, and rfhSP-D-only wells, along with untreated (cells only) cells, were stained after 24 h with Annexin V/FITC; the quantification of apoptosis was carried out using flow cytometry (Figure 6A). Addition of HA to rfhSP-D reduced apoptosis induction by ~45%. Since we noticed a reduced apoptosis induction by HA+ rfhSP-D, we considered if rfhSP-D induced a proliferative response in combination with HA. Thus, HA + rfhSP-D-treated breast cancer cells were stained with mouse anti- Ki-67 antibody to detect the percentage proliferation (Figure 6B). Ki-67 is a well-known nuclear protein associated with active cell proliferation and is expressed throughout the active cell cycle, including G1, S, G2, and M phases. Addition of HA in rfhSP-D-treated SKBR3 cells resulted in ~30% cell proliferation (Figure 6B), suggesting that HA negated pro-apoptotic effects of rfhSP-D in breast cancer cell lines. In the case of BT474, only ~47% of proliferative cells were seen among rfhSP-D-treated BT474 cells, while HA + rfhSP-D-treated BT474 cells showed a higher proliferation (~95%) (Figure 6B). HA-only-treated BT474 cells showed ~88% of Ki-67-positive cells, while ~66% of proliferative cells were seen in HA-treated SKBR3 cells. However, proliferation of BT20 cells was not affected by rfhSP-D or HA treatment, suggesting that these cells continue to grow. These percentage proliferations are compared to untreated control (cells only). Given the presence of SP-D in the breast cancer tissues, we made an attempt to purify SP-D from the culture supernatants of the breast cancer cells on the assumption that breast cancer cells can also be a likely source of SP-D in the tumor microenvironment. We could purify SP-D only from BT474 cell culture (Figure 6C). However, the purified SP-D was not able to induce apoptosis in BT474 cells (Figure 6D).

### rfhSP-D Treatment Upregulated Cell Cycle Inhibitors and Induced p53-Dependent Apoptosis

**Figure 7:**
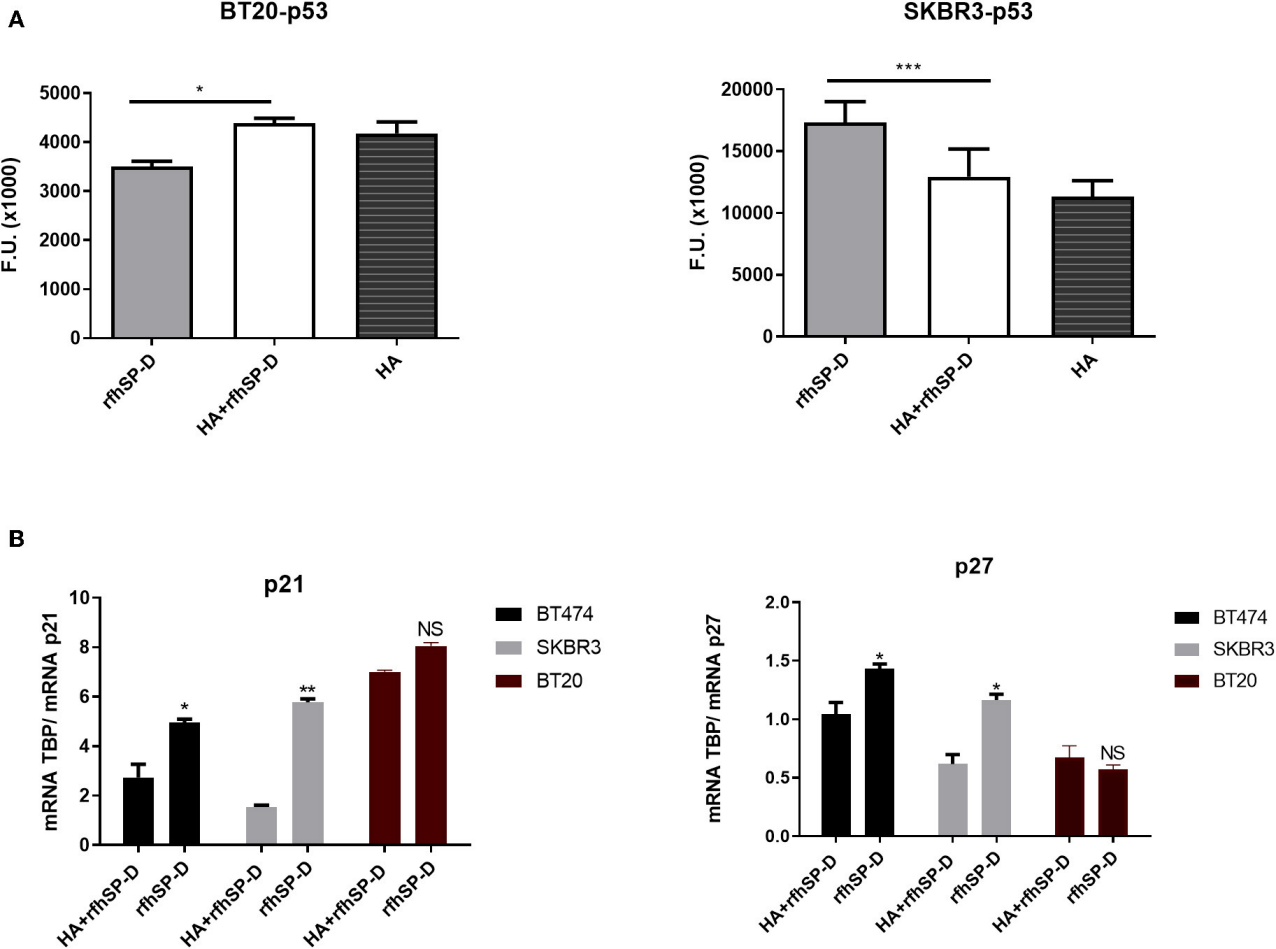
Intracellular signaling to show phosphorylation of p53 in rfhSP-D-treated BT20 and SKBR3 cell lines **(A)**. Breast cancer cell lines were allowed to adhere to HA or HA-bound rfhSP-D, and the phosphorylation status of p53 was evaluated using total cell lysates with a PathScan Antibody Array Kit (Cell Signaling). Data were generated from at least three independent experiments and presented as mean ± SD. rfhSP-D treatment resulted in upregulation of p21 and p27 cell cycle inhibitors **(B)**. BT20, BT474, and SKBR3 (0.4 × 10^6^) cells were seeded in a six-well-plate pre-coated with rfhSP-D and HA + rfhSP-D. The treated cells were lysed and pelleted down. The pelleted cells were subjected to RNA isolation, followed by cDNA synthesis and qPCR. The comparative quantification method was performed to calculate the efficiencies of each gene for each individual PCR and is based on the second differential maximum method or takeoff analysis. The takeoff results obtained with p21/p27 primers were normalized with the housekeeping gene TBP. qPCR assay was conducted in triplicates, and error bars represent ± SEM. Unpaired one-way ANOVA test was used to determine the significance; *p < 0.05, **p < 0.01, and ***p < 0.001 (*n* = 3). The statistical analysis was performed between rfhSP-D and HA + rfhSP-D-treated breast cancer cells.

Activated p53 is a known mediator of DNA damage, oxidative burst, cell cycle arrest, and induction of apoptosis. Therefore, we analyzed the levels of activated p53 in rfhSP-D-treated breast cancer cells. Increased levels of p53 phosphorylation at Ser15 was detected in rfhSP-D-treated SKBR3 cells (Figure 7A), while downregulation was seen in the BT20 cell line (Figure 7A), suggesting that p53 upregulation may also have contributed to p53-dependent apoptosis induction in SKBR3 cells. In addition, increased transcript levels of p21 and p27 were observed in rfhSP-D-treated BT474 and SKBR3 cell lines (Figure 7B) at 12-h time point, suggesting the possibility of cdc2–cyclin B1 reduction leading to G2/M cell cycle arrest. Reduced levels of phosphorylated p53, caspase 3, p21, and p27 were observed in HA + rfhSP-D-treated SKBR3 and BT474 cells (Figures 7A,B), suggesting reduced apoptosis induction by the addition of HA. In the case of BT20, no significant difference was found in terms of p21 and p27 mRNA expression levels between rfhSP-D- and HA + rfhSP-D-treated BT20 cells.

## Discussion

In this study, we show that rfhSP-D binds to all breast cancer cell lines tested: BT20 (ER^−^/PR^−^/HER2^−^), BT474 (ER^+^/PR^+^/HER2^+^), and SKBR3 (ER^−^/PR^−^/HER2^+^). However, the receptor, SP-D is binding to on the cancer cell surface, is not yet known. Exogenous treatment with rfhSP-D reduced cell viability of BT474 and SKBR3 cell lines and induced apoptosis at 24 h. Cell viability and apoptosis assays were carried out using varied concentrations of rfhSP-D (5, 10, and 20 μg/ml) and different time points (12, 24, and 48 h), but only 20 μg/ml concentration of rfhSP-D was effective in inducting the optimal cell viability reduction and apoptosis induction at 24 h. rfhSP-D-treated BT474 and SKBR3 cells showed a significant reduction in viable cells compared to untreated control (cells only), as evident from Trypan blue exclusion and MTT assay (data not shown). However, no effect of rfhSP-D was seen on the triple-negative (BT20) cell line in terms of cell viability. Recent studies have shown the ability of the CRD region of human SP-D to interact with N-glycans of EGFR, resulting in downregulation of EGF signaling in A549 cell line (23). It is likely that rfhSP-D-mediated apoptosis is due to HER2 expression on BT474 and SKBR3 cells; a lack of it seems to increase BT20 cells resistant to the rfhSP-D effect. Whether HER2 is implicated in mediating rfhSP-D-induced apoptosis is under investigation.

The ability of rfhSP-D to induce apoptosis at 24 h in SKBR3 and BT474 cell lines was evident from a significant increase in the number of Annexin V-/PI-positive breast cancer cells examined via flow cytometry and fluorescence microscopy. The cell surface of the healthy cells is composed of asymmetrically distributed lipids on the inner and outer surfaces of the plasma membrane. PS is one of these lipids, which is restricted to the inner surface of the plasma membrane and, thus, is only exposed to the cytoplasm of the cell. However, when apoptosis is triggered by stress factors, lipid asymmetry is lost, and PS translocates to the outer leaflet of the plasma membrane (45). Annexin V is a 36-kDa calcium-binding intracellular protein that binds to PS (45). Thus, Annexin V can also stain cells undergoing necrosis due to a ruptured membrane that permits the access of Annexin V to the entire plasma membrane. Western blotting analysis of rfhSP-D-treated BT474 and SKBR3 breast cancer cells revealed cleavage of caspases 9 and 3 at 12-h treatment. Caspases are well-known cysteine-aspartic proteases that play a crucial role in apoptosis; caspase 9 is triggered by cellular stress (e.g., DNA damage), via its binding to apoptotic protease-activating factor 1 (Apaf-1). Apaf-1/procaspase 9, in turn, triggers executioner caspases 3 and 7 (46). Caspase 3 is an essential apoptotic executor, triggered by endoproteolytic cleavage at Asp175, which leads to inactivation of PARP (a known marker of apoptosis) (45, 47). These findings indicated that rfhSP-D-mediated apoptosis induction in SKBR3 and BT474 cell lines is likely to occur via the intrinsic apoptotic pathway (48). Recently, an integral role of rfhSP-D in innate immune surveillance against prostate cancer has been reported. rfhSP-D-mediated apoptosis has been suggested to take place by two distinct mitochondrial apoptotic mechanisms (19). It is also possible that p53 upregulation in rfhSP-D-treated BT474 and SKBR3 cell lines may lead to downregulation of the pAkt pathway, resulting in increased levels of Bad and Bax, triggering the release of cytochrome c, caspase 9 cleavage, and apoptosis induction. Furthermore, previous studies have also reported the interaction of SP-D with HMGA1, CD14, CD91–calreticulin complex, SIRPα, and EGFR (19, 22, 23, 49). The involvement of SP-D with these key molecules is likely to be relevant as a part of a possible mechanism/receptor through which rfhSP-D is likely mediating its apoptotic functions on breast cancer cells. As reported in previous studies, the CRD of SP-D can interact with pattern recognition receptors TLR-2, TLR-4 (50), and CD14 (22), which may block pro-inflammatory and pro-survival downstream signaling on breast cancer cells. However, further investigation is crucial to understand the involvement of CRD and its possible interacting partners involved in triggering the downstream mechanism of apoptosis induction.

During the development of breast tumorigenesis, the tumor cells interact with surrounding stroma to create a tumor microenvironment, supporting the growth, survival, and invasion of cancer cells. The ECM is an essential constituent of the niche, leading to progression of malignant tumor formation and proliferation. ECM is composed of a wide range of proteoglycans and glycosaminoglycans, which offers a structural support and promotes tissue organization, thus contributing to cell survival, proliferation, invasion, angiogenesis, and immune cell infiltration (35, 51). HA is one of the specific components of ECM, which is synthesized as a large linear anionic polymer at the cell surface. The role of HA during inflammation-mediated breast cancer has been established (52, 53). *In vitro* studies have reported the ability of HA to regulate tumor cell migration and invasion and tumor growth and progression using *in vivo* models (39). Synthesis and accumulation of HA have been detected in invasive breast cancer cells compared to normal breast epithelial tissues (39). Additionally, HA synthase 2 facilitates invasion in breast cancer cells (51). Furthermore, tumor cells overexpressing HA synthase 2 have been shown to enhance angiogenesis and stromal cell recruitment, suggesting that increased levels of HA in the tumor stroma support breast carcinoma (51). Pro-tumorigenic roles of increased HA expression in breast tumors and possible mechanisms through which HA might trigger tumor proliferation are areas of great interest. Therefore, we examined the interaction between rfhSP-D and HA and its implication on the adhesion and proliferation of BT20, BT474, and SKBR3 breast cancer cells.

Direct binding ELISA revealed that rfhSP-D bound to HA in a dose-dependent manner. The ability of breast cancer cells to interact with HA-bound rfhSP-D was examined via a cell adhesion assay. Cell adhesion assay was performed to assess the effect of rfhSP-D or HA treatment on the ability of breast cancer cells to adhere. A greater cell adhesion binding pattern was observed with rfhSP-D alone, when compared to HA-bound rfhSP-D or HA alone. This may be due to the interaction of rfhSP-D-treated cells with apoptotic-related receptors and pathways leading to apoptosis induction. Apoptotic and proliferative assays were also performed to investigate the effect of HA-bound rfhSP-D in comparison to rfhSP-D alone. SKBR3 cells seeded on HA-bound rfhSP-D resulted in an increased cell proliferation (1.7-fold), when compared to rfhSP-D alone. In the case of rfhSP-D-treated BT474 cells, addition of HA resulted in a two-fold increase of proliferative cells. However, the percentage of proliferating cells in the presence of HA-bound rfhSP-D was not significantly different from that in HA alone or untreated SKBR3 and BT474 cells. Therefore, these findings suggest that pro-apoptotic effects of rfhSP-D are modulated by HA addition. However, there was no significant difference in the proliferation of BT20 cells among all the conditions, suggesting that these cells continue to grow and are not affected by rfhSP-D treatment alone or in conjunction with HA. It is also possible that addition of HA may modulate the interaction of rfhSP-D-treated cells with pro-apoptotic signaling pathways, thereby restricting the possibility of apoptosis induction. Thus, addition of HA could facilitate cell proliferation by interacting with proliferative signaling cascades (e.g., Ras-ERK and PI3K-Akt). Even though breast cancer tissues express SP-D (Figures 4i,ii), the presence of HA within the breast tumor microenvironment seems to provide a self-protective coat, thus negating the anti-tumorigenic properties of SP-D. Since BT474 and SKBR3 cells treated with rfhSP-D undergo apoptosis and addition of HA to rfhSP-D enhances cell proliferation, it is likely that breast cancer cells can use HA in their tumor microenvironment as an escape mechanism from apoptosis induction triggered by rfhSP-D.

To further understand the mechanism of apoptosis induction by rfhSP-D treatment, the likely signaling pathways involved in tumor adhesion, proliferation, and cell death were analyzed. rfhSP-D treatment resulted in increased levels of p53 phosphorylation (at Ser15), possibly mediated by oxidative stress caused by rfhSP-D (17, 49). p53 is an essential transcriptional modulator of apoptosis and plays a crucial role in cellular response to DNA damage as well as other genomic alterations. Phosphorylated p53 and increased levels of P21/P27 expression may cause inactivation of cyclin B–cdc2 complexes that are crucial in regulating G2/M cell cycle transition, causing DNA repair or induction of apoptosis (17). However, decreased levels of caspase 3 and p53 were detected in HA + rfhSP-D-treated cells, possibly suggesting its resistance to apoptosis by addition of HA. Although the p21 trigger is p53 dependent in certain events such as DNA damage, there are several scenarios in which p21 expression is independent of p53 status, such as normal tissue development and differentiation. p21 can lead to tumor evolution directed toward tumor growth by slowing down the accumulation of DNA damage. Induction of p21 expression has been shown to be crucial for promoting motility of tumor cells and tumorigenesis. Due to controversial roles of p21, it can be considered as an oncogenic protein and/or tumor suppressor, depending on its cytoplasmic localization. The dual roles of p21 as an oncogenic or tumor suppressor make it difficult to study the mechanisms involved in p53–p21-mediated apoptosis induction or cancer progression.

Targeting HA synthesis, accumulation, degradation, or HA–receptor interaction represents a potential therapeutic approach for treatment of breast and other cancers. The use of 4-MU to inhibit HA synthesis has been shown to reduce breast cancer proliferation and migration (54–57). 4-MU treatment has also been shown to inhibit intracellular and cell surface HA (57), in addition to suppression of Akt phosphorylation. Use of hyaluronidases to degrade HA in the tumor microenvironment has also been considered as a potential therapeutic approach for cancer. Bacteriophage hyaluronidase has been shown to effectively inhibit growth, migration, and invasion of breast cancer cells (58). Hyaluronidase treatment has also shown promising outcomes in pancreatic cancer in clinical trials (59–61). Furthermore, recombinant human hyaluronidase (rHUPH20) has been shown to improve drug delivery of antibody-based trastuzumab targeted therapy in HER2^+^ breast cancer (62). A recent study has shown that HA degradation by pegvorhyaluronidase (PVHA) can remodel the TME in a murine model of breast cancer so as to increase the uptake of anti-Programmed Death-Ligand 1 (PD-L1) therapeutic antibody. PVHA treatment coincided with a dramatic effectiveness of anti-PD-L1 antibody in reducing the tumor growth (63).

Involvement of rfhSP-D and its associated pro-apoptotic properties in cancer models might be a novel therapeutic agent to target multiple cellular signaling pathways, including tumor cell survival factors, transcriptional factors, protein kinases, and pro-apoptotic gene signatures. The mechanism which enables rfhSP-D to induce apoptosis and inhibit tumor cell proliferation is tumor specific due to the differential effects on the cell types and probably expression of putative receptors. The nature of interaction between HA + rfhSP-D and cellular receptors is currently under investigation. The function of SP-D in the biology of cancer is complex and is strongly dependent on the tumor microenvironment. As SP-D and HA are overexpressed in breast cancer and other carcinomas, targeting both HA and SP-D is clinically relevant to inhibit HA-mediated intracellular signaling that negates the pro-apoptotic role of rfhSP-D. It is important to note that FL-SP-D purified from BT474 was not able to induce apoptosis in any of the three breast cancer cell lines. The plausible reasons could be that the tumor cells produce a dysfunctional protein owing to mutations, protein misfolding, and/or altered oligomerization.

In conclusion, rfhSP-D treatment was able to induce apoptosis in BT474 and SKBR3 cell lines involving the intrinsic apoptotic pathway, while having no effect on the triple-negative BT20 cell line. In addition, HA-bound rfhSP-D was able to restore cell growth, suggesting that these breast cancer cells may use HA as an escape mechanism to overcome pro-apoptotic effects of rfhSP-D. The mechanisms by which HA-bound rfhSP-D restores cell proliferation are yet to be elucidated.

## Data Availability Statement

The raw data supporting the conclusions of this article will be made available by the authors, without undue reservation.

## Ethics Statement

Surgical breast cancer tissues and adjacent peritumoral mammary parenchymas were selected following ethical approval by the University Hospital of Palermo, Italy (approval number 09/2018).

## Author Contributions

Data curation: CA, PV, and TM; formal analysis: HK, TM, and RB; investigation: SA and AK; methodology: VM, PV, BB, and TR; resources: SV; supervision: RB and UK; validation: FA; writing—original draft: VM; writing—review and editing: UK. All authors contributed to the article and approved the submitted version.

## Conflict of Interest

The authors declare that the research was conducted in the absence of any commercial or financial relationships that could be construed as a potential conflict of interest.
